# An investigation into the avoidability of adverse drug reactions using the LAAT and modified Hallas tools

**DOI:** 10.1097/MD.0000000000018569

**Published:** 2020-01-03

**Authors:** Mohammed Ibn-Mas’ud Danjuma, Shaikha Al Shokri, Ibrahim Y. Abubeker, Ashraf El Malik, Ibtihal Mahmoud Hassan Abdallah, Mohamed Nabil El Shafei, Haajra Fatima, Mohamed Mahmoud, Tanweer Hussain, Yahya Maghoub, Jamal Sajid, Abdel Naser El Zouki

**Affiliations:** a Weill Cornell Affiliated-Hamad General Hospital, Hamad Medical Corporation; Weill Cornell Medicine; College of Medicine, Qatar University; b Division of General Internal Medicine, Weill Cornell Affiliated-Hamad General Hospital, Hamad Medical Corporation; c Unity Internal Medicine Rochester Regional Health New York; d Division of General Internal Medicine, Weill Cornell affiliated-Hamad General Hospital, Hamad Medical Corporation, Doha, Qatar; Acute Medicine Unit, Derbyshire Hospitals Foundation Trust, Derby, England, United Kingdom; e Emergency Department, Weill Cornell affiliated-Hamad General Hospital, Hamad Medical Corporation,; f Weill Cornell Affiliated-Hamad General Hospital, Hamad Medical Corporation; g Clinical Pharmacist, Weill Cornell affiliated-Hamad General Hospital, Hamad Medical Corporation,; h Weill Cornell Affiliated-Hamad General Hospital, Hamad Medical Corporation; i Weill Cornell affiliated-Hamad General Hospital, Hamad Medical Corporation, Doha, Qatar; j Wirral University Teaching Hospital NHS Foundation Trust, Liverpool, United Kingdom; k Weill Cornell Affiliated-Hamad General Hospital, Hamad Medical Corporation; l Hamad General Hospital, Hamad Medical Corporation (HMC), Doha, Qatar, Department of Clinical Sciences, College of Medicine, Qatar University, Weill Cornell Medical College of Qatar, Doha, Qatar.

**Keywords:** adverse drug reactions, agreement, avoidability, reliability

## Abstract

An adverse drug reactions avoidability tool called the Liverpool ADR avoidability assessment tool (LAAT) was recently developed (for research purposes), and subsequently validated with mixed interrater reliability (IRR). We investigated the comparative IRR of this tool in an inpatient cohort to ascertain its practical application in this setting.

The patient population was comprised of 44 ADR drug pairs drawn from an observational prospective cohort of patents with ADR attending a Weill Cornell Medicine-affiliated tertiary medical Centre in Doha Qatar (Hamad General Hospital). Using the LAAT, and modified Hallas tools, 4 independent raters (2 Clinical Pharmacologists, and 2 General Physicians) assessed and scored the 44 ADR-drug pairs. Agreement proportions between the rating pairs were evaluated as well individual/overall kappa statistics and intraclass correlation coefficients. We evaluated the weight of each of the 7 questions on the LAAT tool to ascertain its determinative role.

Across 44 ADR-drug pairs, the overall median Fleiss kappa using the LAAT, and modified Hallas tools were 0.67 (interquartile range (IQR) 0.55, 0.76), 0.36 (IQR, 0.23–0.71) respectively. The overall percentage pairwise agreement with the LAAT and modified Hallas tools were 78.5%, and 62.2% respectively. Exact pairwise agreement occurred in 37 out of 44 (range 0.71–1), and 27 of 44 (0.53–0.77) ADR-drug pairs using the LAAT and modified Hallas tools respectively. Using the LAAT tool, the overall intraclass correlation coefficient was 0.68 (CI 0.55, 0.79), and 0.37 (CI 0.22, 0.53) with the modified Hallas tool.

We report a higher proportion of “possible” and “definite” avoidability outcomes of adverse drug reactions compared with the modified Hallas, or that reported by developers of the LAAT tool. Although initially developed for research purposes, our report has suggested for the first time a potential applicability of this tool in clinical environment as well.

## Abstract 2

Supplemental Digital Content is available in the text

**Key Points**

- We found a higher proportion of inter-rater reliability (IRR) with the LAAT tool compared with that reported by developers of the tool.
- We have demonstrated the first attempt at potential clinical applicability of the LAAT tool.
- There is potential for improvement of the LAAT tool especially questions that rely on imputation
- Good Knowledge of clinical therapeutics is a determinant of demonstrating ADR avoidability.
- The LAAT tool have improved on the methodological flaws of the modified Hallas tool.

## 1 Introduction

Adverse drug reactions (ADR)continue to contribute significantly to admission burden in emergency rooms, as well as accounting for morbidity and mortality amongst hospitalized patients.^[1,2]^ The overall incidence of ADR amongst hospitalized patients is estimated at 6% to 14.7%.^[3,2]^ Recognizing the principal drug classes involved is amongst the most important steps towards reducing this burden. ADR's could be inadvertent due to known and unknown sequelae of drugs. In an increasing proportion however, the cause is very often due to drug error with potentially preventable time course.^[4]^ The prevalence of preventable adverse drug reaction is variable but it is estimated to contribute significantly to the overall proportion of reported ADR's.^[4]^ As result of this, new ADR adjudication concepts have developed in the whole narrative of ADR, that is, the concept of preventability or avoidability of ADR.^[5]^

Consequent upon this, clinical algorithms and scoring systems have since been developed aimed at estimating the preventability of specific ADR's, with the view to reducing their overall burden on clinical therapeutics.^[1,6,7]^ The modified Hallas algorithm remains the most widely used and studied tool amongst these tools.^[1]^ Despite this, the inconsistency seen with studies evaluating ADR avoidability still persist largely due to lack of standardization of terminologies amongst others.^[4,8]^ The Liverpool ADR avoidability tool (LAAT) was therefore recently developed to especially mitigate some of the adjudicating flaws previously reported with the modified Hallas tool.^[8]^ These includes subjective requirement for user of the tool to have a comprehensive knowledge of the underlying pathology and details about its optimal treatment amongst others.^[8]^ The LAAT tool which has since been validated initially for research purposes, have a better user interface with identifiable information available to the user. It has thus far reported mixed inter-rater agreement (IRR).^[8]^ How this tool performs in a clinical setting of patient population with significant polypharmacy remains unknown. In this study we have investigated the comparative capacity of determining avoidability of ADR between the “gold-standard” (modified Hallas tool), and the LAAT tool in a distinct population of inpatients. Additionally, deconstructing the path of assessment of Liverpool causality assessment tool (LCAT) tool has been recently studied to ascertain the determinative role specific questions within the LCAT tool contribute in arriving at the tool's ordinal outcomes.^[9]^ To our knowledge, no such evaluation of the LAAT avoidability tool has been reported, and it will be interesting to ascertain which of the seven questions contributes most to the adjudicating process. It will additionally be useful to determine if this tool could be restructured based on the relative contribution of each of the 7 questions to adjudication of ADR avoidability.

## 2 Methodology

All consecutive patients presenting with suspected ADR to the either the acute admissions unit or ED or are inpatients of the Weill Cornell Medicine affiliated-Hamad Medical corporation, Doha, Qatar were recruited into the study cohort as part of a prospective observational ADR cohort. From these cohort, we selected a random sample of 10 cases for training of ADR avoidability raters on assessing avoidability including familiarity with the tools. A further random sample of N = 44 cases were designated as the study cohort and used for ADR avoidability assessment. Relevant demographic and clinical variables of each patient was abstracted from an online patient record system (Cerner), and transferred unto a study specific excel database. Variables abstracted includes age, gender, self-declared ethnicity, list of current medication, history of drug allergies, past medical history, index ADR, suspected drug (s) involved, date ADR drug commenced, date ADR drug stopped, date of onset of symptoms/signs, duration of ADR symptoms and signs, details of investigations for alternative or differential diagnosis of the suspected ADR, outcome of ADR, any record of re-challenge. Where there was need for further clarification, we arranged interviews with the patients concerned. We included all ADR cases excluding cases of intentional drug overdoses. A prior request for ethical approval was submitted to the independent review board of the Medical research Centre (HMC). This was considered and ethical approval provided before commencement of the study.

Four reviewers, (2 Clinical Pharmacologists, 2 General Physicians) independently assessed, and scored the ADR-drug pairs using the modified Hallas and LAAT tools for avoidability along the 4 ordinal outcomes. Utilizing patient demographic, clinical, laboratory data of individual ADR cases, each rater assessed causality of ADR using the LCAT tool, and subsequently determined ADR avoidability using both LAAT and Hallas tools. ADR avoidability outcomes were reported as “definitely avoidable”, “possibly avoidable”, “not avoidable” and “unassessable”. The distribution of the specialty of cases considered is shown in Table 1. Where raters require additional information for ADR adjudication, we advised accessing internationally recognized clinical guidelines or recommendations from statutory therapeutic regulatory agencies such as the food and drug administration (FDA), medicines and healthcare products regulatory agency (MHRA) or the European medicine agency (EMEA). Additional therapeutic information sources include summary of product characteristics (SmPC), specialty guidelines, and British national formulary.

**Table 1:**
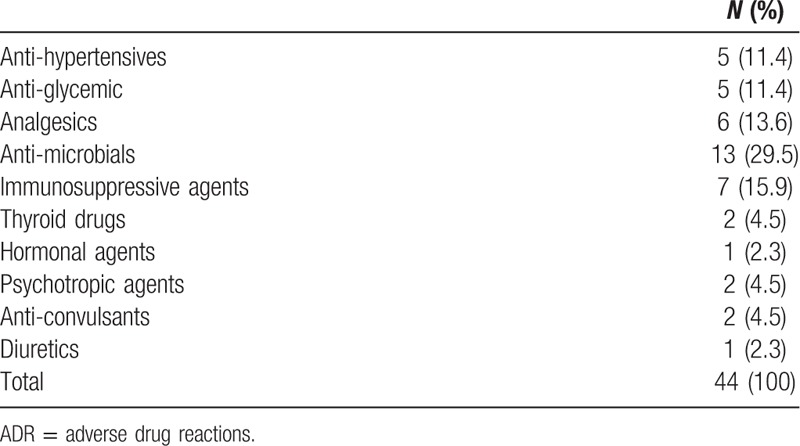
Distribution of drug classes associated with ADR-drug pairs.

## 3 Statistical analyses

Avoidability outcomes are represented as categorical variables, with their pairwise interrater agreement proportions, Fleiss kappa statistics with 95% confidence intervals (CI), and intraclass correlation coefficients (ICC). To determined agreement across multiple assessors we calculated and compared the pairwise scores with a global kappa score.

## 4 Case definitions

- We confirm extreme agreement (EA) between 2 raters if they both score an ADR-drug pair to the same outcome using the same tool^[10]^
- Extreme disagreement (ED) was defined as a situation where a rater adjudicates an ADR-drug pair outcome as unassessable whilst the other rater assessed it as any of the three other outcomes (“not avoidable”, “possibly avoidable”, or “definitely avoidable”).
- We estimated pairwise agreements with, and without inclusion of cases adjudicated as “unassessable”. Such cases are treated as missing values. Results are thus presented as Krippendorf's alfa (because of its ability to handle missing values).
- Kappa values of ≤0.20, 0.21 to 0.40, 0.41 to 0.60, 0.61 to 0.80, and 0.81 to 1 correspond to slight, fair, moderate, substantial, and almost perfect agreement, respectively^[11]^

## 5 Results

The mean age of the study cohort was 43.3 (±17.3), with a high proportion of male population (N = 29; 65.9%). Antimicrobial drugs accounted for about 15% of the ADR-drug pairs (Table 1 gives a breakdown of other drug classes). Using the LAAT tool, the proportion reported as “avoidable” (“possibly” and “definitely”) was 39.8% (N = 70) (Table 2).

**Table 2:**
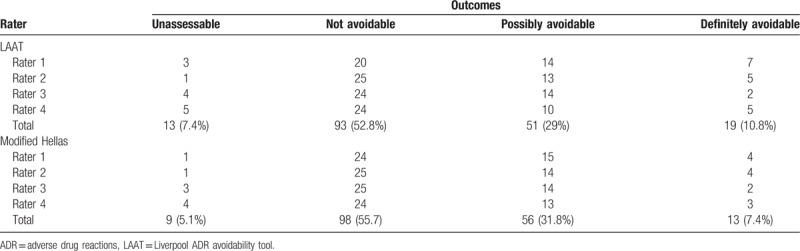
Distribution of ADR avoidability outcomes using LAAT and modified Hellas tools.

## 6 Disposition of avoidability outcomes

The LAAT tool resulted in total of 176 drug-ADR outcome decisions (across the 2 rating pairs), out of which 13 were adjudicated as “Unassessable” (7.4%), 93 as “not avoidable” (52.8%), 51 “possibly avoidable” (29%), and 19 “definitely avoidable” (10.8%). The modified Hallas tool resulted in 9 “assessable” (5.1%), 98 “not avoidable” (55.75), 56 “possibly avoidable” (31.8%), and 13 “definitely avoidable” (7.4%) outcomes (Table 2).

## 7 Inter-rater agreement and reliability

The overall Median Fleiss kappa using the LAAT, and modified Hallas tools were 0.67 (interquartile range (IQR) 0.55, 0.76), 0.36 (IQR, 0.23–0.71) respectively. The overall percentage pairwise agreement with the LAAT and modified Hallas tools were 78.5%, and 62.2% respectively.

Exact pairwise agreement occurred in 37 out of 44 (range 0.71–1), and 27 of 44 (0.53–0.77) ADR-drug pairs using the LAAT and modified Hallas tools respectively (Table 3). The proportion of exact agreement (EA) amongst clinical pharmacologists, and physician rating pairs using the LAAT and modified Hallas tools were 0.9 (39 of 44 cases), and 0.78 (35 of 44 ADR cases) respectively. Extreme disagreement (ED) occurred in 12 of 44 cases with the LAAT tool, and 7 of 44 ADR drug pairs with the modified Hallas tool.

**Table 3:**
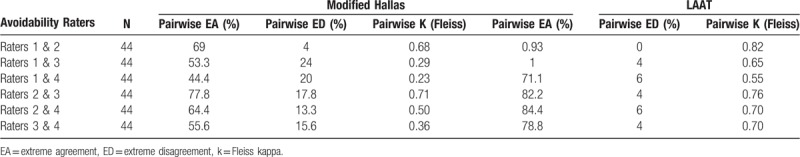
percentage pairwise Fleiss kappa agreement/disagreement in the assessment of avoidability between LAAT and modified Hallas tool.

Using the LAAT tool, the overall intraclass correlation coefficient was 0.68 (CI 0.55, 0.79), and 0.37 (CI 0.22, 0.53) with the modified Hallas tool.

We removed and classified cases adjudicated by the raters as “unassessable” as missing values, the overall ordinal Krippendorf's alpha before and after exclusion of these cases were the same for the LAAT and modified Hallas tools 0.64 and 0.39, respectively.

## 8 The path of determination of avoidability

**Figure 1:**
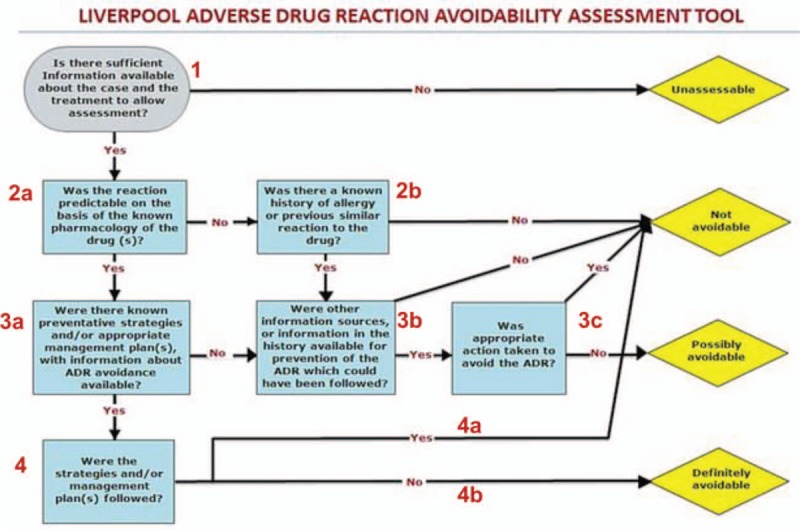
A numbered version of the Liverpool adverse drug reactions avoidability tool. The seven determinant questions have been numbered to determine the “path of arrival” at the 4 ordinal outcomes (“unassessable”, “not avoidable”, possibly avoidable”, “definitely avoidable”). Adapted from^[8]^

We investigated the path taken by each of the 2 rating pairs in arriving at the 4 ordinal outcomes. We observed that of the 4 possible paths of arriving at a “not avoidable” outcome, the path with the designation “1–2a-3a-3b” has the highest frequency (see Fig. 1 for labelling of the LAAT tool). The path “1–2a-3a-4a” was the least frequent (Table 4). There was no possibility of investigating the “possibly avoidable” outcome path as this follows a single path (1–2a-3a-3b-3c-No). All ADR raters reported lack of clarity regarding specialty guidelines from which reliable information that may assist in answering question “3a” on the LAAT tool could be obtained (supplemental file [Fig. 1]).

**Table 4:**
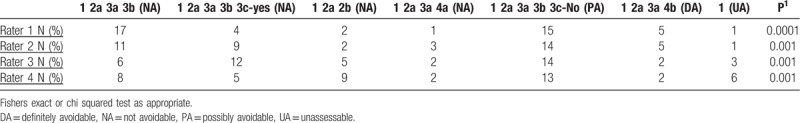
Path to determination of avoidability using the LAAT tool.

## 9 Discussion

To our knowledge, this is the first study to attempt exploration of the potential clinical utility of the recently validated LAAT tool for the determination of ADR avoidability amongst cohorts of adult patients presenting with ADR. Examination of ADR-drug pairs using this tool showed that about 38% were avoidable (“possibly” and “definitely”), with a substantial inter-rater reliability (median pairwise kappa agreement of 0.67, IQR 0.55, 0.76). This is consistent with the ballpark range reported by developers of the tool (Kappa 0.12–0.75).^[8]^ Similarly, we found a higher percentage of extreme agreement (EA) with the LAAT tool (37/44 cases, range 0.71–1) compared with the modified Hallas tool (24/44 cases, range 0.53–0.77). Since its validation, the percentage of EA reported with the LAAT tool range between 25% to 70%.^[8]^ The flaws inherent in the modified Hallas tool have exhaustively been discussed elsewhere,^[8,12]^ but the judgmental nature of some of the questions perhaps account for some of the lower EA scores seen in our study using this tool. Overall, we found the LAAT tool to have a higher interrater reliability compared with the modified Hallas tool.

We report a higher proportion of “definitely” avoidable outcomes compared with the developers of the LAAT tool. The higher percentage of EA seen with both the LAAT and modified Hallas tools in our study compared with that reported by Bracken et al^[8]^ may probably have to do with differences in the study methodology, and patient populations amongst a range of other factors. The LAAT tool was derived from a pediatric demographic population with a preponderance of oncology cases, whereas our patient ADR-drug pair cohort spans the entire length of clinical therapeutics. The “inevitability” associated with some of the oncology ADRs (such as neutropenia with chemotherapy) makes the adjudication of these ADRs as “not avoidable” a more likely outcome. These may have accounted for the relatively higher proportion of avoidable (“possibly” and “definitely”) in our study population amongst other factors. These includes the use of fewer raters in our study (4) compared to Bracken et al.^[8]^

Although the LAAT tool was developed for the determination of avoidability of research ADR cases, we explored its utility amongst a range of clinical ADR cases to ascertain utility in identifying a cohort of these patients that intervention strategies could potential work on. This is consistent with the long term prospects of the developers of the LAAT tool, who also envisage its applicability perhaps in other settings other than research.^[8]^ The determination of ADR avoidability is increasing seen as a critical factor in the overall scheme targeting ADR sub-types that could be amenable for intervention by both drugs and therapeutic committees as well as regulatory bodies. Amongst the key principles expounded by the LAAT tool is the identification and adherence to best management practices as the fulcrum of ADR avoidability.^[8]^ Others includes presentation of a tool whose interface was made up of reasonable and readily available information clinicians and prescribers could access in the course of their daily practice.^[8]^ This is in contrast markedly to the absolute reliance on clinical judgment from previous tools, including the Modified Hallas tools.^[4,6]^

Additionally, we found a higher percentage of EA and IRR (39/44 cases) reported by the Clinical Pharmacologist rating pairs compared with Physicians (34/44 cases). This may perhaps be a factor of better access to available therapeutic information which clinical pharmacologist by the nature of their training will most probable have over and above those of General Physicians.

There is currently no validated method or framework of training for use of the ADR adjudication and avoidability tools. To enhance operability of the tools, we randomly assigned 10 cases each to the 2 rating pairs to familiarize themselves with the use of the tools over a week period. This was designated as a training cohort.

The range of ADR-drug pairs we adjudicated for avoidability span the length and breadth of various organ systems including cardiovascular, antimicrobial and immunosuppressive drugs. The developers of the LAAT tool had expressed concern regarding the potential validity of the tool given the diversity and varying robustness of clinical guidelines from which reliable clinical management information could be gleaned from. All ADR raters reported issues with question “3a”, perhaps the mostly likely of the 7 questions to be open to different interpretation (depending on access relevant clinical information/ guidelines). Our observation was that except where guideline recommendations regarding management are explicit, there is the potential for clinical imputation to play a significant role in the determination of this question (“3a”).

We investigated the path taken by each rater in scoring and arriving at the 4 ordinal outcomes, this is to identify questions and or “paths” that could impact on the validity of the tool. We found the path “1–2a-3a-3b” to be the most frequent amongst all raters out of the 4 possible paths leading to a “not avoidable” outcome. Despite multiplicity of paths leading to “not avoidable” outcome, we found no compelling evidence of jettisoning any of the other least frequent paths. This is so because we investigated if any of the questions along the least frequent paths were judgmental, and apart from the issues highlighted above with question “3a”, we found this not be the case.

## 10 Limitation

Our study may have been limited by the lack of standardization of the training of the raters regarding the use of the avoidability tools. Training of raters is an essential part of mitigating some of the flaws of modified Hallas tool as reported by previous studies.^[4]^ To date however, there has not been any agreement on the most optimal duration of training, most of what has been reported thus far is self-guided training. Additionally, what level of Kappa scores represent the optimal threshold for IRR in the adjudication of ADR-drug pair avoidability remains unknown. The developers of the LAAT tool suggested assigning a “lower” Kappa level because of the original intention of the tool for research purposes, rather than clinical case adjudication. The lack of complete patient clinical information as observed by some of the raters may have impacted on the robustness of the avoidability adjudication process

## 11 Conclusion

We report a higher proportion of “possible” and “definite” avoidability outcomes of adverse drug reactions compared with the modified Hallas, or that reported by developers of the LAAT tool. Although initially developed for research purposes, our report has suggested a potential applicability of this tool in clinical environment as well.

## Acknowledgment

We would like to acknowledge the contribution of Mostafa Eldesoukey who assisted with study patient recruitment.

## Author contributions

**Conceptualization:** Mohammed Ibn-Mas’ud Danjuma, Ibrahim Yusuf Abubeker, Abdelnaser Awad El Zouki.

**Data curation:** Mohammed Ibn-Mas’ud Danjuma, Shaikha Daoud Na Al Shokri, Ibrahim Yusuf Abubeker, Tanweer Hussain, Ashraf Malik, Ibtihal Mahmoud Hassan Abdallah, Nabil Abdelsalam El Shafei, Haajra Fatima, Yahya Mahgoub, Jamal Sajid.

**Formal analysis:** Mohammed Ibn-Mas’ud Danjuma, Shaikha Daoud Na Al Shokri, Ibrahim Yusuf Abubeker, Tanweer Hussain, Ashraf Malik, Ibtihal Mahmoud Hassan Abdallah, Nabil Abdelsalam El Shafei, Haajra Fatima, Mohamed Ibrahim Mohamed Mahmoud, Yahya Mahgoub, Abdelnaser Awad El Zouki.

**Funding acquisition:** Mohammed Ibn-Mas’ud Danjuma, Abdelnaser Awad El Zouki.

**Investigation:** Mohammed Ibn-Mas’ud Danjuma, Ibrahim Yusuf Abubeker, Ibtihal Mahmoud Hassan Abdallah, Nabil Abdelsalam El Shafei, Haajra Fatima, Mohamed Ibrahim Mohamed Mahmoud, Yahya Mahgoub.

**Methodology:** Mohammed Ibn-Mas’ud Danjuma, Shaikha Daoud Na Al Shokri, Ibrahim Yusuf Abubeker, Tanweer Hussain, Ashraf Malik, Ibtihal Mahmoud Hassan Abdallah, Nabil Abdelsalam El Shafei, Haajra Fatima, Mohamed Ibrahim Mohamed Mahmoud, Yahya Mahgoub, Jamal Sajid, Abdelnaser Awad El Zouki.

**Project administration:** Mohammed Ibn-Mas’ud Danjuma.

**Resources:** Mohammed Ibn-Mas’ud Danjuma, Ibrahim Yusuf Abubeker, Yahya Mahgoub.

**Software:** Mohammed Ibn-Mas’ud Danjuma, Ibrahim Yusuf Abubeker, Yahya Mahgoub.

**Supervision:** Mohammed Ibn-Mas’ud Danjuma, Mohamed Ibrahim Mohamed Mahmoud.

**Validation:** Nabil Abdelsalam El Shafei, AbdelNaser Awad El Zouki.

**Writing – original draft:** Mohammed Ibn-Mas’ud Danjuma, Shaikha Daoud Na Al Shokri, Ibtihal Mahmoud Hassan Abdallah, Jamal Sajid.

**Writing – review & editing:** Mohammed Ibn-Mas’ud Danjuma, Yahya Mahgoub, Abdelnaser Awad El Zouki.

Mohammed Ibn-Mas’ud Danjuma orcid: 0000-0003-2198-5278.

## Supplementary Material

Supplemental Digital Content
